# Methodological Critique of Concussive and Non-Concussive Dual Task Walking Assessments: A Scoping Review

**DOI:** 10.3390/ijerph20065227

**Published:** 2023-03-22

**Authors:** Courtney Jade Mitchell, John Cronin

**Affiliations:** 1 Sport Performance Research in New Zealand (SPRINZ), AUT Millennium Institute, AUT University, Auckland 1010, New Zealand; 2 Department of Sport and Recreation, Toi Ohomai Institute of Technology, Tauranga 3112, New Zealand; 3 Athlete Training and Health, 23910 Katy Freeway, Suite 101, Katy, TX 77494, USA

**Keywords:** concussion, gait, locomotion, dual task

## Abstract

Objective: To understand the methodological approaches taken by various research groups and determine the kinematic variables that could consistently and reliably differentiate between concussed and non-concussed individuals. Methods: MEDLINE via PubMed, CINAHL Complete via EBSCO, EBSCOhost, SPORTDiscus, and Scopus were searched from inception until 31 December 2021, using key terms related to concussion, mild traumatic brain injury, gait, cognition and dual task. Studies that reported spatiotemporal kinematic outcomes were included. Data were extracted using a customised spreadsheet, including detailed information on participant characteristics, assessment protocols, equipment used, and outcomes. Results: Twenty-three studies involving 1030 participants met the inclusion criteria. Ten outcome measures were reported across these articles. Some metrics such as gait velocity and stride length may be promising but are limited by the status of the current research; the majority of the reported variables were not sensitive enough across technologies to consistently differentiate between concussed and non-concussed individuals. Understanding variable sensitivity was made more difficult given the absence of any reporting of reliability of the protocols and variables in the respective studies. Conclusion: Given the current status of the literature and the methodologies reviewed, there would seem little consensus on which gait parameters are best to determine return to play readiness after concussion. There is potential in this area for such technologies and protocols to be utilised as a tool for identifying and monitoring concussion; however, improving understanding of the variability and validity of technologies and protocols underpins the suggested directions of future research. Inertial measurement units appear to be the most promising technology in this aspect and should guide the focus of future research. Impact: Results of this study may have an impact on what technology is chosen and may be utilised to assist with concussion diagnosis and return to play protocols.

## 1. Introduction

Concussions are a mild traumatic brain injury (mTBI) that individuals can experience through sport that are frequently missed or underestimated resulting in individuals returning to sport earlier than they should, increasing the risk of sustaining a musculoskeletal injury [1,2] or leading to further brain damage if a second concussion is experienced in close proximity to the first event [3,4]. There is a need to have protocols that can assess the extent of the concussion experienced while also determining readiness for return to activity. Typical methods of assessing concussions are clinical assessments which consider physical and mental attributes such as balance and memory, respectively [5]. These assessments are generally tested as two separate elements, yet researchers have suggested that a dual task (DT) assessment that combines physical and mental testing provides a more accurate understanding of concussion than standalone walking and cognitive assessments [6,7,8,9,10].

Protocols incorporating a cognitive task alongside a gait assessment are becoming frequently utilised to evaluate the effects of concussion [11,12,13]. The Stroop test, which involves participants responding to an auditory or visual cue whilst undergoing locomotion, requires equipment to facilitate the test and record the accuracy and speed of responses [6,13,14,15,16,17,18,19,20]. Other more common cognitive dual tasks include reciting the months of the year in reverse order, subtracting by sixes or sevens from a given number, or spelling common five letter words in reverse, while walking along a level walkway [7,11,21,22,23,24,25,26,27,28,29,30,31,32]. Gait variables such as walking speed, stride length and cadence are quantified to determine any variability through the introduction of a cognitive task [11,12,23,32,33].

To measure the physical variables, 3D motion capture (3D MOCAP) and/or force plates are commonly used to differentiate between concussive diagnoses by assessing postural balance and control [6,7,9,11,14,15,16,21,22,23,24,25,26,27,28,30,34]. Inertial measurement units [8,31,32,33,35,36] and accelerometers [18] are other forms of technology that have been used for DT concussion gait analysis. However, whether certain technologies and/or certain variables are better suited to discriminating between concussed and non-concussed diagnoses is unknown. Of particular interest to the authors and that which provides the purpose of this scoping review was understanding the methodological approaches taken by various research groups and determining those variables that could consistently and reliably differentiate between concussed and non-concussed individuals.

## 2. Methods

### 2.1. Data Sources and Searches

**Figure 1:**
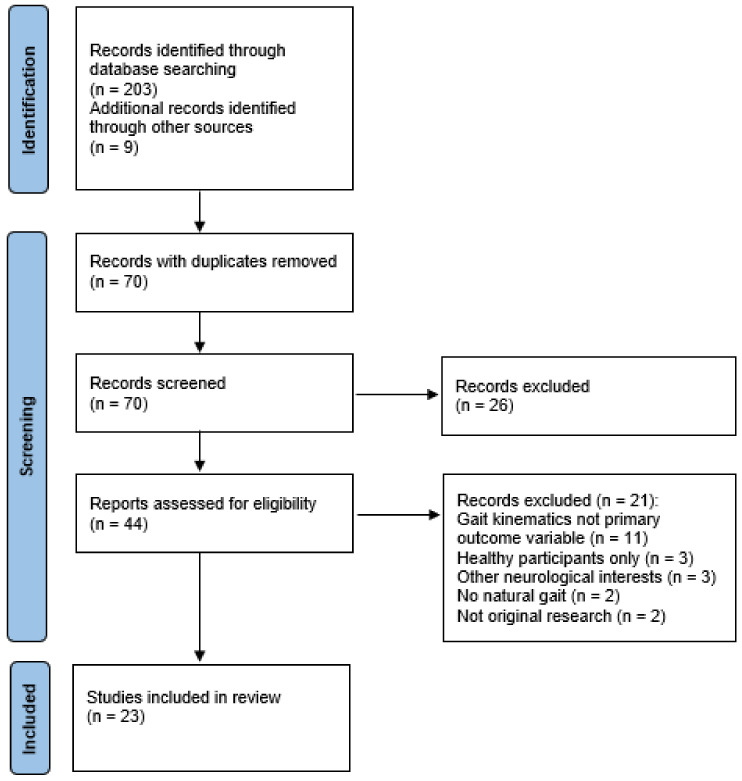
PRISMA-ScR flow diagram of the study selection process. The flow of information through the review phases is depicted, detailing the number of articles that were included and excluded in the review, with reasons for exclusions.

A scoping review was conducted guided by the standards presented by the Preferred Reporting Item for Systematic Reviews and Meta-Analysis extension for Scoping Reviews (PRISMA-ScR) [37]. This review aimed to examine the methodological approaches, determine those variables that could consistently and reliably differentiate between concussed and non-concussed individuals, identify limitations of current technologies and related protocols and, finally, to outline areas of future research for concussion assessment. MEDLINE via PubMed, CINAHL Complete via EBSCO, EBSCOhost, SPORTDiscus and Scopus databases were searched for relevant articles from the inception of the databases until 31 December 2021. The search strategy included five concepts (concussion, mTBI, cognition, gait and dual task) and a combination of key words to adapt to each database. From the initial screening, 70 articles were identified, and titles and abstracts were screened to determine relevance to review. Forty-four full-text articles were examined to determine inclusion eligibility. Reference lists of included articles were searched for other potentially relevant information. A total of 23 articles were identified as being eligible for full-text review and subsequent analysis (Figure 1).

### 2.2. Study Selection

Studies were included if a steady state DT walking assessment was used; the DT involved a cognitive task paired with a steady state gait task; individuals had a concussion, either through sport or other activity, or mild traumatic brain injury with healthy individuals used as control subjects; and kinematic walking measures were reported. There were no restrictions placed on the age or gender of participants. Articles were excluded if steady-state gait was not the primary dependent variable of cognitive task performance, such as reaction time, tandem gait, balance or gait termination time. Review articles and case studies were excluded. Full-text articles were retrieved and scanned when inclusion could not be determined by screening titles and abstracts. Articles that involved all healthy participants or those with a more severe brain injury were excluded from the analysis. Risk of bias was mitigated in this research, given the focus was more a technological/methodological critique rather than a review of the outcome measures and findings as such.

### 2.3. Data Extraction

One reviewer (C.M.) extracted the data using a customised designed standardised Excel database (version 2201, Microsoft, Redmond, WA, USA) which was validated by a secondary reviewer (J.C.). General study information (i.e., author, year), subject characteristics (i.e., sample size, age, concussion history, sport/activity), type of study (i.e., cross-sectional and prospective), methods of assessment (i.e., testing equipment, environment, protocol) and primary outcome measures (e.g., means and standard deviations of average gait velocity) were extracted. Descriptive information relating to the sport and performance level were used to categorise each of the participants. A wide array of definitions for elite, sub-elite and novice athletes exist (30). Therefore, in order to clearly differentiate between groups with concussions, skill level was grouped according to the level at which participants were competing. National or regional representatives were classified as elite athletes. Participants competing at university or collegiate (Uni/Col) were categorised as sub-elite. Recreational athletes were deemed as such (Rec) and those who did not experience a sport-related concussion were classified as NoSport. Adolescent athletes were categorised as high school (HS) athletes.

### 2.4. Role of the Funding Source

This scoping review was funded by Movement Solutions. The funder played no role in the design, collation, synthesis and writing of this review.

## 3. Results

### 3.1. Study Characteristics

Eight of the twenty-three articles (35%) employed prospective designs, with assessments at two to five time points over the course of the study [6,7,11,18,24,25,33,38]. Fifteen articles (65%) utilised cross-sectional designs [12,13,15,16,20,21,22,23,28,31,32,36,39,40,41]. The total number of participants used across the studies was 1030, with 474 participants categorised as concussed subjects and 556 participants categorised as non-concussed controls. Due to the lack of detail provided, it is unclear as to whether there was any repeated usage of sample groups. Grade II concussion parameters described by the American Academy of Neurology (AAN) were detailed in seven articles [11,21,22,23,24,25,28] and seven articles used the latest Consensus Statement on Concussion in Sport (CsoCiS) [6,7,18,31,32,38,40]. One article used the Veteran Health Affairs/Department of Defence mTBI criteria for concussion diagnosis [36] and seven articles did not state the method of concussion diagnosis [13,15,20,33,39,41]. Concussions were diagnosed by certified athletic trainers and/or medical professionals in 17 articles [6,7,11,12,13,15,21,24,25,28,31,32,33,38,39,40,41]; however, the remaining six did not state who diagnosed the concussions of the participants [16,18,20,22,23,36].

### 3.2. Participants

From the 1030 participants, 54% were male and 49% were female; the sex of the participants was not reported in one study [25]. There was insufficient detail provided in each of the studies to determine if there was any overlap of sample groups. The age of participants most commonly ranged between 13 and 22, often being high school and university students and athletes. One study involved adults over 64 years of age [39]. The majority of concussions experienced by participants were sport-related, with the sporting level ranging from elite athletes, intercollegiate athletes, and high school athletes to local and recreational athletes. Three studies included subjects who had sustained a concussion through activities of daily living [24,25,38]. Subjects experienced concussion through a variety of sports with the most common sports reported being football, American football and ice hockey (*n* = 2) [31,33].

### 3.3. Gait Protocol

The description of the gait protocols can be seen in Table 1. The most common distance covered for the protocols was between 8 and 10 m (*n* = 11 [11,12,13,15,21,22,24,25,28,33,39]). All 23 articles (100%) involved participants walking at a self-selected pace, 14 of which had participants walking barefoot [6,7,11,18,21,23,24,25,31,32,33,36,38,40]. Testing locations were described as a laboratory (*n* = 6) [11,12,13,15,20,23], walkway or hallway (*n* = 12) [6,7,18,21,22,24,25,31,33,38,39,41] or were unspecified (*n* = 5) [16,28,32,36,40]. The most frequent number of trials used across the articles was five trials per testing condition (*n* = 9) [11,12,20,21,25,28,32,39,40], followed by between eight and ten trials (*n* = 4) [6,7,15,38]. The amount of practice trials varied from one [18], four [13,15] and “several” [22,23,28]. In most circumstances (17 articles–74%) it was not stated whether any practice or familiarisation took place. Inter-trial rest periods were described as being 30 s [12] and “several minutes” [23]; however, for the most part (91% of articles) the rest periods were not detailed. Level walking as a single-task (ST) assessment protocol was used in 21 studies [6,7,11,12,13,15,16,20,21,22,23,24,25,28,31,32,33,36,38,39,41]; two articles did not include a ST assessment [18,40]. There was considerable variation in the number of testing occasions for each study: eight articles had one testing occasion up to 72 h [21,23,28], 5–7 days [20,31,32,40] or 4–15 weeks [41] post-concussion; three articles included four testing occasions at time points of up to 72 h, 5–7 days, 2 weeks and 1 month post-concussion [11,24,25]; five testing occasions were utilized in four articles at time points of up to 72 h, 5–7 days, 2 weeks, 1 month and 2 months post-concussion [6,7,18,38]; one article [33] had two testing occasions with the initial occasion up to 21 days post-concussion and the second occasion occurring once no symptoms were being experienced; one article incorporated one testing occasion and did not detail how soon participants were recruited following a concussion being experienced [22]; six articles had one testing occasion, where participants had experienced a concussion during their lifetime (“history of concussion”) [12,13,15,16,36,39].

### 3.4. Cognitive Task

Eight different cognitive tasks were utilised in the DT gait protocols across the 23 studies (see Table 1). These included: spelling a common five letter word backwards; subtracting by sixes and/or sevens; reciting the months of the year in reverse; auditory Stroop; visual Stroop; and Brooks’ spatial memory task, verbal fluency, and arithmetic. The most commonly used DT cognitive tasks (*n* = 14) were spelling common five letter words backwards, subtracting by sixes and/or sevens and reciting the months of the year in reverse [6,11,16,20,21,22,23,24,25,28,31,32,33,40,41]. An auditory Stroop assessment was the next most common cognitive task (*n* = 6) [6,7,18,20,36,38], followed by a visual Stroop test [13,15,16] and a verbal fluency task [13,16,41] (both *n* = 3). All 23 studies included at least one DT assessment. In terms of the number of cognitive tasks used within each methodology, a single DT assessment was used in seven articles [6,12,15,18,36,38,39], two different DT tests were used in two articles [20,41], three different cognitive tasks were used in six articles [7,11,13,16,24,25] and eight articles randomised participants’ single DT trial from three DT options [21,22,23,28,31,32,33,40].

### 3.5. Equipment

The equipment used in the articles reviewed can be observed in Table 2. Motion capture (3D MOCAP) was used in 16 articles [6,7,11,13,15,16,18,20,21,22,23,24,25,28,38,39]. The number of markers placed on bony landmarks mostly ranged between 25 and 32 (*n* = 12) [6,7,11,18,21,22,23,24,25,28,38,39], with one group of researchers utilising 16 markers [20] and three research groups using four markers [13,15,16]. The number of cameras used ranged between six and ten (*n* = 12) [6,7,11,16,18,21,22,23,24,25,28,38]; however, four research groups did not state how many cameras were used [13,15,20,39]. The most widely used sampling rate was 60 Hz (*n* = 10) [6,7,11,18,21,22,23,25,28,38], where other researchers sampled data at 100 Hz [15], 120 Hz [20] and 240 Hz [39]. The sampling rate was not stated in three papers [13,16,24]. Marker trajectory data was filtered using a low-pass fourth order Butterworth filter by 11 research groups (69%), with a cut-off filter of 6 Hz [13,15] and 8 Hz [6,7,11,16,21,22,23,25,28,38] being the most common cut off frequencies. The method of data filtering was not stated in five articles [16,18,20,24,39].

Nine research groups utilised force plates in conjunction with 3D MOCAP [6,7,11,21,22,23,24,25,38]; two in-series force plates were used in six articles [11,21,22,23,24,25] and three articles used three in-series force plates [6,7,38]. A sampling rate of 960 Hz was used in all but two articles [6,7,11,21,22,23,25,38], with the sampling rate not being specified in these studies [20,24].

Inertial measurement units (IMU) were utilised by five research groups [31,32,33,36,40]. IMUs were placed on the lumbosacral junction and dorsum of each foot (*n* = 4) and recorded data at a sampling rate of 128 Hz [31,32,33,40]. One article placed IMUs on the dorsum of each foot, forehead, lumbar spine and sternum, with the sampling rate not being specified [36]. A single research group utilised an accelerometer in combination with 3D MOCAP [18]. The accelerometer was attached at the L5 vertebrae and collected data at a sampling rate of 128 Hz.

Three articles used a microphone to record participants’ responses during their respective DT [6,7,15]. A GAITRite walkway, sampling at 80 Hz, was used to collect gait data in one article [12]. One article utilised a manual stopwatch to time participants’ gait [41].

### 3.6. Outcome Measures

The outcome measures of interest are detailed in Table 3. Gait velocity was the most studied measure in terms of identifying concussive gait impairments. No significant differences in gait velocity across all monitored time periods were reported in ten articles [6,12,13,15,20,21,24,25,39,41], whereas significant differences were reported in 13 articles [7,11,16,18,22,23,28,31,32,33,36,38,40]; the most common differences were found with concussed individuals having a slower gait velocity at <72 h after injury (*n* = 7) [7,11,18,22,23,28,38] and 5–7 days after injury (*n* = 5) [7,18,31,32,40]. Concussed subjects had a slower gait velocity in four articles [6,7,21,24], yet this difference was not enough to be considered significant.

In terms of the stride/step parameters (length, time, width), stride/step length seemed to be the more sensitive of the measures, with six out of 12 research groups reporting significant differences between concussed and non-concussed gait [11,21,32,33,36,40]. Significant differences in stride/step length were reported at <72 h post-concussion (*n* = 2) [11,21], 5–7 days (*n* = 2) [20,32], 2 weeks (*n* = 2) [11,33] and with historic concussions (*n* = 2) [33,36]. Five research groups utilised stride time, with two groups reporting significant differences at <72 h post-concussion [22,23] and one group reporting significant differences with historic concussion [36]. All eight of the articles that reported stride/step width measures found no significant differences [6,11,12,21,22,23,28,39].

Regarding cadence and double support, there was a paucity of researchers investigating the sensitivity of these measures over time, with double supporting having largely been discussed with historic concussion subjects only (*n* = 4) [12,33,36,39]. Two out of four articles which included double support analysis found a significant increase in double support duration for historically concussed individuals [12,36].

Of the five articles that used IMUs to differentiate between concussed and non-concussed subjects, significant differences were reported regarding gait speed (*n* = 5) [31,32,33,36,40], stride length (*n* = 4) [32,33,36,40], cadence (*n* = 2) [32,33], stride time (*n* = 1) [36] and double support (*n* = 1) [36].

### 3.7. Reliability

None of the studies reviewed established the reliability of the specific protocols they implemented. Four of the articles reviewed referred to reliability of the equipment and protocols established in other studies (Table 4). On reviewing these studies, two research groups investigated the reliability of GAITRite walkway variables, which only one reviewed article used [12]. Montero-Odasso et al. [42] considered gait velocity, step length, stride length, step time, stride time and double support time in single and dual task walking with a cognitively impaired elderly population (average age 76.6 ± 7.3 y). The absolute consistency (coefficient of variation (CV)) ranged from 6.36–18.28% for ST and 11.02–19.27% for DT. In terms of relative consistency, intraclass correlation coefficient (ICC) ranged from 0.80–0.97 for ST and 0.93–0.97 for DT. The GAITRite walkway was also investigated by Paterson et al. [43], however, the comparison was between younger (20.08 ± 0.7 y) and older (67.93 ± 7.8 y) populations. CVs ranged from 2.33–4.08 %. In terms of relative consistency, ICCs ranged from 0.66–0.94.

The GAITRite walkway was also utilised in conjunction with inertial sensors to establish reliability of other technologies using continuous walking protocols. Moore et al. [44] sought to establish the reliability of a wearable accelerometer (AX3) with stroke patients. Within the variables of step velocity, step length, step time, and stance time, the absolute agreement was good (ICC: 0.744–0.797) between AX3 and GAITRite, and moderate–excellent (ICC: 0.831–0.923) between AX3 and Opal inertial sensors. Morris et al. [45] compared GAITRite with Opal inertial sensor data analysed via Mobility Lab across young adults, older adults and adults with Parkinson’s disease. Gait velocity, stride length, cadence and stride time had moderate–excellent absolute agreement (ICC: 0.741–0.998); however, double support time had poor absolute agreement (ICC: 0.213–0.716).

To establish reliability of cognitive tasks while walking, Howell et al. [30] used IMUs to investigate the ST and DT gait of collegiate athletes in both contact and non-contact sports (19.2 ± 1 y) through gait speed, cadence and stride length. This research group only reported relative consistency: the ICCs ranged from 0.68–0.80 for ST and 0.73–0.85 for DT walking.

## 4. Discussion

Concussions are an increasingly common mild traumatic brain injury that are experienced in sport. To limit misdiagnosis of individuals with concussion and to assist with return to play, there is a need for assessment protocols that incorporate both cognitive and physical elements to allow for a more accurate evaluation of concussive impairment. Assessing gait whilst performing a cognitive task is one such assessment protocol and formed the focus of this review. Of particular interest were the methodological approaches taken by various research groups and determining those protocols and/or variables that could consistently differentiate between concussed and non-concussed individuals.

The participants involved across the reviewed articles were diverse in sample size (12–122), age (12–68 y), sport (football, cheerleading, horseback riding, to name a few) and competition level (recreational–elite). Sixty-one percent of the reviewed study protocols required participants to partake barefoot, which presents an interesting issue in terms of whether testing should take place with shoes or barefoot, which potentially may affect the clinical outcomes. Counting or spelling backwards seemed to be the easiest of dual tasks to implement given the ease of administration and lack of equipment required, negating the need for extensive set up time. It is suggested that these cognitive tests should be randomised to limit any learning effects.

The most widespread use of equipment involved 3D MOCAP and force plates. While the equipment may be considered to provide more precise information, the cost of the equipment and the expertise required to run, process and analyse the data is a restrictive factor for assessing concussions outside of conducting research. A significant time cost is also involved with processing the information recorded from MOCAP and force plates to generate data for analysis. Equipment that does not require as extensive proficiency or time to process and analyse collected data, such as with inertial sensor technology, may offer a more accessible tool for practitioners in diagnosing and monitoring concussion.

The most common distance that participants were assessed over with dual task gait was 8–10 m. This was largely a result of the space in which the testing was conducted and the available equipment e.g., 2–3 force plates in series and/or in ground with 3D MOCAP. The authors feel that the set-up of such equipment is a limitation, in that testing is restricted to a particular environment (i.e., sports laboratory) which may impede the initial diagnosis and subsequent monitoring of concussed individuals, thus, being detrimental for quicker return to play. More portable technologies (i.e., IMUs) may provide a more accessible and convenient tool that can be utilised within a wide range of environments. If dual task gait analysis of concussive diagnosis is to have any real-world utility, then serious consideration of other technological approaches will be needed.

None of the 3D MOCAP and force plate outcome measures reported were found to be sensitive enough to consistently determine differences between concussed and non-concussed diagnosis during DT walking. Gait velocity, stride/step length and stride/step width were the variables that were most reported on, with significant differences being reported by 31% [11,18,22,23,38] and 25% [11,21] of the reviewed articles for gait velocity and stride/step length, respectively, but no article was found to report significant differences in stride/step width. The majority of articles found no significant differences across the gait variables of interest. Comparatively, articles that utilised IMUs to measure gait velocity and stride/step length reported significant differences in 100% [31,32,33,36,40] and 80% [32,33,36,40] of the articles, respectively. This may indicate that IMU utilisation enables increased accuracy and/or sensitivity due to a closer interaction with the gait movement patterns. It also needs to be noted that the diagnostic value of any gait analysis is enhanced when data is collected over multiple testing occasions. This historic data provides a better insight into any aberrations that may need addressing.

The emergence of inertial sensor technology [31,32,33,36,40] might provide a viable alternative to MOCAP and force plate analyses. The outcome variables reported by the articles that utilised IMUs showed promising consistency in differentiating between concussed and non-concussed diagnoses. It would be interesting to understand whether different sensor placements (e.g., in-sole sensors) offer added sensitivity and accuracy, compared to the sensor arrangements in the bulk of the studies reviewed (lumbosacral, dorsum and foot).

One of the most concerning aspects of all the articles reviewed was the absence of any reporting of the reliability of the outcome measures of interest. Understanding the “noise” or unexplained variability associated with a measure is fundamental to interpreting findings. Only one research group [12] provided evidence regarding the reliability of the GAITRite walkway in elderly and young cohorts, citing the work of Paterson et al. (2008) [43] and Montero-Odasso et al. (2009) [42]. The results were markedly different in that Paterson et al.’s [43] findings were acceptable (CV < 4.08%; ICCs 0.66–0.94), whereas the absolute consistency of Montero-Odasso et al. [42] was not (DT CVs 11.02–19.27%; ICCs 0.93–0.97). This could be attributed to the age of the participants in the latter study. Nonetheless, it needs to be noted that only Martini et al. [12] used the GAITRite walkway as a method of measuring DT gait variables and, therefore, it is problematic to make generalisations to other methodological approaches.

## 5. Conclusions

However, whether certain technologies and/or variables are better suited in discriminating between concussed and non-concussed diagnoses is unknown.

Of particular interest to the authors was understanding the methodological approaches taken by various research groups and determining those variables that could consistently and reliably differentiate between concussed and non-concussed individuals. In terms of the first foci, MOCAP and force plates were the dominant technologies used to quantify concussed and non-concussed gait. From the literature reviewed, it would seem that none of the gait parameters assessed using MOCAP and force plates used to quantify concussed and non-concussed gait impairments were consistently sensitive enough to determine significant differences between groups, particularly over various time periods/testing occasions. This may mean two things: (1) DT walking is not sufficiently sensitive enough as an assessment to determine concussive diagnosis consistently; or (2) the protocols/technologies that are being used need refining or replacing to enable better concussion detection. For example, it would be interesting to determine if longer distances/large fields of capture enabled better precision of measurement.

With regards to the consistency and reliability of data, there seems to be little attention in the research reviewed on the variability of the measures utilised to quantify gait characteristics. Fundamental to research going forwards, especially with new and innovative technology, is establishing the reliability and smallest worthwhile changes in gait parameters.

Inertial sensor technology has been used in a few studies to date with some promising results around average gait speed and stride length. However, as with the other technologies reviewed, the reliability has not been documented and there may be better placement of sensors than the lumbar and dorsum but researchers have provided a starting point for ongoing investigation. For example, it would be interesting to determine if inertial sensors that quantify the foot–ground interaction (e.g., inner sole sensors) offer any diagnostic benefits in this area.

Finally, the cost of MOCAP and force plates and the expertise required to run, process and analyse the data is a restrictive factor for assessing concussions outside of conducting research. It is believed that the advent of technological “solutions” such as inertial sensors may enable dual task testing outside of the laboratory given the portability of such devices. If the technology is found to be valid, reliable, accurate and sensitive to changes in gait characteristics, they may provide a viable assessment option that could result in higher utility of dual task walking assessments in the diagnosis of concussion.

## Figures and Tables

**Table 1:** Sample groups and protocols from reviewed studies.

Author (Year) Sample Size Age Concussion Classification Sport	Protocol Description
Parker et al. (2005) [21] *n* = 20 C *n* = 10, 6 F, 4 M 20.20 ± 1.7 y NC *n* = 10, 6 F, 4 M 19.90 ± 1.9 y Concussion Classification: MP AAN Grade II Uni/Col and Rec	ST: walk down a 10 m level walkway at a comfortable self-selected pace while barefoot. DT: spelling five letter words in reverse, subtraction by sevens and reciting months of the year in reverse order. DT randomly selected for each walking trial Testing began with five trials of ST, followed by the DT condition. C referred for testing as soon as possible after injury.
Parker et al. (2006) [11] *n* = 30 C *n* = 15, 6 F, 9 M 20.60 ± 1.6 y NC *n* = 15, 6 F, 9 M 20.60 ± 1.8 y Concussion Classification: MP AAN Grade II Uni/Col and Rec	ST: walk down a 10 m level walkway at a comfortable self-selected pace while barefoot. DT: spelling five letter words in reverse, subtraction by sevens and reciting months of the year in reverse order. DT order rotated across trials. Each session began with five trials of ST walking followed by four to five trials of DT. All C were tested within 48 h post injury and 5, 14 and 28 days post injury. NC tested at similar intervals. Testing conducted in laboratory.
Catena et al. (2007) [22] *n* = 28. University students C *n* = 14, 6 F, 8 M 22.3 ± 4.5 y NC *n* = 14, 6 F, 8 M 22.3 ± 3.1 y Concussion Classification: AAN Grade II MP Sport not stated.	ST: walk down an 8 m walkway at a self-selected pace. DT: QA = spelling a common five letter word in reverse; continuous subtraction, reciting months of year in reverse. Order of tasks not shared prior to testing. Several practice trials allowed to ensure familiarity and foot contact was made with both force plates.
Catena et al. (2007) [23] *n* = 28 C *n* = 14, 6 F, 8 M 22.29 ± 4.5 y NC *n* = 14, 6 F, 8 M 22.29 ± 3.1 y Concussion Classification: AAN Grade II Who diagnosed not stated Sport not stated	No ST; walking component of DT: walk down an 8 m walkway at a comfortable self-selected pace while barefoot. DT 1: QA = spelling a common five letter word in reverse, continuous subtraction by a certain number and reciting the months of the year in reverse order. Several practice trials, ensuring whole foot contact made with force plate. Each participant performed approx. 60 trials. Each trial lasted approx. 8 s. Returned to same starting position for each trial. Several minutes rest twice during the testing session during transition to new testing condition. C testing occurred within 48 h post injury. Testing completed in laboratory.
Parker et al. (2007) [24] *n* = 58 C *n* = 29, 14 F, 15 M 21.60 ± 3.3 y NC *n* = 29, 14 F, 15 M 21.38 ± 3.4 y Concussion Classification: AAN Grade II MP Uni/Col and ADL	ST: walk down a 10 m level walkway at a comfortable self-selected pace while barefoot. DT: spelling five letter words in reverse, subtraction by sevens and reciting months of the year in reverse order Order of individual tasks rotated across trials. C tested within 48 h post injury, and at 5, 14 and 28 days post injury. NC tested at same intervals.
Parker et al. (2008) [25] *n* = 56 C athletes *n* = 14 20.71 ± 1.3 y C non-athletes *n* = 14 22.43 ± 4.6 y NC athletes *n* = 14 20.64 ± 1.5 y NC non athletes *n* = 14 22.93 ± 4.3 y Concussion Classification: AAN Grade II MP Uni/Col, Rec, and NoSport	ST: walk down a 10 m level walkway at a self-selected pace while barefoot. DT: spelling five letter words backwards, subtraction by sevens and reciting the months of the year in reverse. Order of individual tasks rotated across trials. Each testing session began with four to five trials of ST walking followed by four to five trials of DT. C tested within 48 h post injury, and at 5, 14 and 28 days post injury. NC tested at same time intervals.
Martini et al. (2011) [12] *n* = 68 C *n* = 28, 11 F, 17 M 21.00 y NC *n* = 40, 20 F, 20 M 21.72 y Concussion Classification: MP and self-reported Sport not stated	ST: walking along GAITRite walkway (0.89 × 8.3 m) at a self-selected pace. DT: Brooks’ Spatial Memory Task–verbally recite the spatial location of digits 1 through 8 in an imaginary 4 × 4 grid. Participants recall position of numbers while walking. 24 unique grids presented randomly. Conditions: ST walk, DT walk. Each condition ×5, in a random order. 30 s rest between trials Tested in research laboratory.
Fait et al. (2013) [15] *n* = 12 C *n* = 6, 2 F, 4 M 19.7 ± 2.3 y NC *n* = 6, 2 F, 4 M 20.1 ± 2.7 y Concussion Classification: MP How diagnosed not stated Elite athletes	ST: straight unobstructed walk 8.75 m at a comfortable walking pace without stopping. DT: modified visual Stroop Four level walking trials conducted first to establish walking speed. A baseline for the Stroop word task was performed (two 5 s trials). Overall, two gait conditions combining the different walking Stroop (with or without) tasks. Five trials of each condition performed in a random order Testing conducted in laboratory
Howell et al. (2013) [6] *n* = 40 C *n* = 20, 2 F, 18 M 15.3 ± 1.3 y NC *n* = 20, 2 F, 18 M 15.6 ± 1.0 y Concussion Classification: CsoCiS MP HS athletes	ST: walk at a self-selected pace while barefoot, along a walkway. DT: auditory Stroop test Eight to ten consecutive trials completed for each ST and DT conditions. C tested within 72 h of sustaining concussion, then 1 week, 2 weeks, 1 month and 2 months post injury. NC tested similar schedule.
Cossette et al. (2014) [16] *n* = 14 C *n* = 7, 6 F, 1 M 20.0 ± 1.6 y NC *n* = 7, 6 F, 1 M 22.4 ± 1.4 y Concussion Classification: Not stated Rec	ST: Walking 6 m at a comfortable and constant speed. DT: - Visual Stroop - Verbal fluency (naming as many words beginning by a given letter) - Arithmetic (counting backward by two from a given number). Four baseline trials initially (level walking no cognitive task); then every combination of conditions repeated four times. Elements of cognitive tasks were changed across trials.
Howell et al. (2014) [7] *n* = 46 C *n* = 23, 3 F, 20 M 15.4 ± 1.3 y NC *n* = 23, 3 F, 20 M 15.7 ± 1.3 y Concussion Classification: CsoCiS MP Sport not stated	ST: walk barefoot at a self-selected pace along a walkway. DT 1: single auditory Stroop DT 2: multiple auditory Stroop = four responses DT 3: QA = spelling a five letter word backwards, subtracting by 6 s or 7 s or reciting months in reverse order. QA randomly selected for each trial. Eight to ten trials of each ST and DT conditions. C tested within 72 h of injury, and again at 1 week, 2 weeks, 1 month and 2 months post injury. NC tested at similar timeline.
Chen et al. (2015) [28] *n* = 60 C *n* = 15, 6 F, 9 M 21.3 ± 3.3 yrs NC *n* = 15, 6 F, 9 M 21.2 ± 3.4 yrs Concussion Classification: AAN Grade II MP Sport not stated	ST: 8 m walk at a self-selected pace. DT: spelling five letter words in reverse, subtraction by sevens and reciting months of year in reverse order. Randomly selected for each trial Several practice trials for participant familiarity Data from five trials collected for each testing condition C referred for testing within 48 h of injury
Howell et al. (2015) [18] *n* = 17 C *n* = 10, 3 F, 7 M 19.0 ± 5.5 y NC *n* = 7, 4 F, 3 M 20.0 ± 4.5 y Concussion Classification: CSoCiS Who diagnosed not stated Sport not stated	No separate ST: barefoot walk at a self-selected pace along a walkway DT: auditory Stroop test. Practice trial first. C tested within 72 h post injury, then 1 week, 2 weeks, 1 month and 2 months post injury. NC tested at similar schedule.
Cossette et al. (2016) [13] *n* = 27 C *n* = 14, 6 F, 8 M 13.0 ± 1.6 y NC *n* = 13, 5 F, 8 M 12.8 ± 1.6 y Concussion Classification: MP Sport not stated	No separate ST: walk 8 m at a comfortable pace DT: - Visual Stroop test - Verbal fluency of naming words, beginning with a given letter - Counting backward by twos from a given number Each session began with four trials of level walking to familiarise participants with the lab. All combinations of locomotor and cognitive tasks were repeated four times.
Martini et al. (2016) [39] *n* = 77 20 years old C *n* = 16, 7 F, 9 M 20 ± 2 y NC *n* = 24, 12 F, 12 M 22 ± 2 y 40 years old C *n* = 4, 1 F, 3 M 47 ± 4 y NC *n* = 15, 8 F, 7 M 45 ± 4 y 60 years old C *n* = 7, 1 F, 6 M 63 ± 4 y NC *n* = 11, 4 F, 7 M 64 ± 4 y Concussion Classification: MP, self-reported Sport not stated	ST: walk along a 10 m walkway at a self-selected pace. DT: Brooks’ Mental Task—remember and recall the order and location of eight sequential numbers in a 4 × 4 grid that were presented via an audio recording. Five trials of four walking conditions.
Berkner et al. (2017) [33] *n* = 81 C *n* = 37, 20 F, 17 M 16.2 ± 3.1 y NC *n* = 44, 25 F, 19 M 15.0 ± 2.0 y Concussion Classification: MP HS/Rec	ST: barefoot level walking at a self-selected pace 8 m to a marker and return to start DT: spelling a five letter word backwards, subtracting 6 s or 7 s from a random 2 digit number or reciting months in reverse from a random month. DT was randomly selected for each trial. C tested at two periods: 1st within 21 days of injury, 2nd after no longer experiencing concussion symptoms. NC only tested once.
Yasen et al. (2017) [38] *n* = 40 C *n* = 20, 10 F, 10 M 21.2 ± 4.4 y NC *n* = 20, 10 F, 10 M 21.4 ± 4.6 y Concussion Classification: CSoCiS MP Rec/ADL	ST: barefoot walking at a self-selected pace along a walkway. DT: auditory Stroop task identifying the pitch of words “high” or “low” spoken in either a high or low pitch. Eight to ten trials completed at each session. Tested at points within 72 h post-concussion, then again at 1 week, 2 weeks, 1 month and 2 months post-concussion. NC tested at similar periods.
Howell et al. (2018) [40] *n* = 59 C *n* = 18, 9 F, 9 M 19.9 ± 1.2 y NC *n* = 41, 12 F, 29 M 19.0 ± 0.9 y Concussion Classification: CSoCiS MP Uni/Col	No separate ST: walking barefoot at a self-selected pace towards a target placed 10 m away, walk around it and return to start position. DT: spelling a five letter word in reverse, subtracting by 6 s or 7 s from a 2 digit number or reverse month recitation. Five trials completed. C tested within 5 days post injury. NC tested as part of baseline testing in preseason.
Solomito et al. (2018) [20] *n* = 31 C *n* = 16, 7 F, 9 M 14.6 ± 1.8 y NC *n* = 15, 6 F, 9 M 13.8 ± 1.4 y Concussion Classification: Not stated Sport not stated	ST: walking at typical pace in a laboratory. DT 1: recite months of the year or days of the week in reverse. DT 2: auditory Stroop test. Cognitive load trials were performed following ST. A minimum of five strides of data were collected per side for each of the three testing conditions. C tested within one week of receiving medical clearance
Howell et al. (2019) [31] *n* = 114 C *n* = 49, 24 F, 25 M 14.9 ± 1.9 y NC *n* = 65, 31 F, 34 M 14.9 ± 1.6 y Concussion Classification: CSoCiS MP HS sport	ST: walk at a self-selected pace to a target placed 8 m in front, walk around, return to start. DT: walking and cognitive. Cognitive: spelling five letter word in reverse, subtracting in 6 s or 7 s from a random two digit number or reciting months of the year in reverse. Randomly chosen. Testing in a quiet hallway, barefoot.
Howell et al. (2019) [32] *n* = 124 C *n* = 54, 25 F, 29 M 20.3 ± 1.1 y NC *n* = 60, 22 F, 38 M 18.9 ± 0.7 y Concussion Classification: CSoCiS MP Uni/Col	ST: walking barefoot at a self-selected pace, to a target 8 m away, around it, and back to start position. DT: spelling five letter word in reverse, subtracting in 6 s or 7 s from a two digit number, or reverse month recitation. DT test rotated between trials to minimise learning effects. Five trials completed in each condition, mean from five trials used for analysis. C tested within 5 days of injury. NC tested as part of preseason testing.
Gagne et al. (2021) [41] *n* = 40 C *n* = 20, 10 F, 10 M 22.1 ± 3.0 y NC *n* = 20, 10 F, 10 M 22.55 ± 2.7 y Concussion Classification: MP Sport not stated	ST: walk back and forth 10 m for a total of 40 m at a natural and comfortable pace. DT: counting backwards in 7 s, producing different words beginning with a specific letter. Instructed to perform the cognitive task as fast and accurately as possible. Tested in a corridor in the rehab institute
Martini et al. (2021) [36] *n* = 122 C *n* = 65, 45 F, 20 M 39.6 ± 11.7 y NC *n* = 57, 36 F, 21 M 36.9 ± 12.2 y Concussion Classification: Veteran Health Affairs Historic Concussion	ST: walking at a comfortable, self-selected pace, eight laps of 13 m path with 180 degree turns at each end. DT: auditory Stroop task identifying the pitch of words “high” or “low” spoken in either a high or low pitch.

Key: n, number of participants; C, concussed subjects; NC, non-concussed subjects; F, female; M, male; y, years; MP, Medical professional; CSoCiS, Current Statement of Concussion in Sport; AAN, American Association of Neurology; HS, high school athletes; Uni/Col, university/collegiate athletes; Rec, recreational athletes; ADL, activities of daily living; ST, single task; DT, dual task; QA, question and answer; m, metres; h, hours.

**Table 2:** Equipment and technologies utilised in reviewed studies.

Author (Year)	Equipment	Variables and Method of Calculation	Testing Protocol Reliability
Parker et al. (2005) [21]	Six camera motion capture ExpertVision HIRES system (Motion Analysis Corporation, Santa Rosa, CA, USA). Twenty-five reflective markers on bony landmarks. Marker trajectory data collected at 60 Hz; low-pass filtered using a fourth-order Butterworth filter, cut off frequency 8 Hz. Motion analysis system calibrated before each session (volume = 4 m long, 1.5 m wide, 2 m high) Two force plates (Advanced Mechanical Technology, Inc., Watertown, MA, USA) in series along gait path, sampled at 960 Hz.	Whole body COM position data calculated as the weighted sum of each body segment (13 segments). COM velocities and accelerations estimated using generalised cross-validated spline algorithm. Motion data analysed from heel strike of the trailing limb as it struck the first force plate to the next heel strike of that same limb. EVa software: estimating virtual marker positions to represent internal segment endpoints from the external markers and the relative positions of segmental COM (Motion Analysis Corporation). External markers and estimated joint centres used to calculate the three-dimensional motion for individual body segments and locations of the segmental COM. OrthoTrak 4.0 (Motion Analysis Corporation) used in the calculation of temporal–distance parameters (gait velocity, stride length, stride time and step width).	None
Parker et al. (2006) [11]	Six camera motion capture ExpertVision HIRES system (Motion Analysis Corporation, Santa Rosa, CA, USA) Twenty-five reflective markers on bony landmarks. Marker trajectory data collected at 60 Hz; low-pass filtered using a fourth-order Butterworth filter, cut off frequency 8 Hz. Motion analysis system calibrated before each session (volume = 4 m long, 1.5 m wide, 2 m high). Two force plates (Advanced Mechanical Technology, Inc., Watertown, MA, USA) in series along gait path, sampled at 960 Hz. Data averaged across trials for each task condition (ST and DT)	Whole body COM position data calculated as the weighted sum of each body segment (13 segments) COM velocities and accelerations estimated using generalized cross-validated spline algorithm. Motion data analysed from heel strike of the limb as it struck the first force plate to the next heel strike of that same limb. EVa software: estimating virtual marker positions to represent internal segment endpoints from the external markers and the relative positions of the segmental COM (Version 6.0, Motion Analysis Corporation). External markers and estimated joint centres used to calculate the three-dimensional motion for individual body segments and locations of segmental COM. OrthoTrak 4.0 (Motion Analysis Corporation) used in the calculation of temporal–distance parameters (gait velocity, stride length, stride time and step width).	None
Catena et al. (2007) [22]	Eight camera motion capture (Motion Analysis Corporation, Santa Rosa, CA, USA). Twenty-nine retroreflective markers bilaterally on bony landmarks. Marker trajectories sampled at 60 Hz for 4 s; filtered through a low-pass, fourth-order Butterworth filter, cut off frequency 8 Hz. Two sequential force plates (Advanced Mechanical Technologies Inc., Watertown, MA, USA) separated by 25.9 cm in centre of walkway; sampled at 960 Hz for 4 s.	Virtual markers created at joint centres and combined with anthropometric data to determine COM location for each of 13 body segments. Motion data calculated for one complete stride; heel strike on to the first force plate to heel strike of the same foot on the second force plate. Whole body COM calculated from each segment COM using a weighted sum method Velocities calculated using Woltring’s generalised cross-validated spline algorithm. Gait velocity: position change of the body COM and time change during a complete stride. Stride length and stride time: position change of the heel marker and respective time change. Step width: left to right ankle joint centres at heel strike.	None
Catena et al. (2007) [23]	Eight Eagle digital cameras positioned surrounding an 8 m walkway (Motion Analysis Corporation, Santa Rosa, CA, USA) Twenty-nine retroreflective markers on anatomical landmarks EVaRT 4.37A collected data at 60 Hz for 4 s; trajectories filtered with a low-pass fourth order Butterworth filter, cut off frequency 8 Hz. Two in-series strain gauge force plates (Advanced Mechanical Technologies Inc. Watertown, MA, USA), in centre of walkway, flush with floor. Data collected at 960 Hz for 4 s. Photocell (RadioShack, Fort Worth Tx, USA) and radio telemetry receiver (TS0611T, Isaacs & Associates Inc., Walla Walla, WA, USA) collected at 960 Hz for 4 s.	COM calculated from 13 different segments. COM calculations based on Dempster’s (Winter 1990) anthropometric data. A weighted sum method used to calculate whole body COM from each segment COM during each time point. COM truncated from first heel strike on to the first force plate to the heel strike of the same foot after the second force plate. COM velocities estimated with Woltring’s generalised cross-validated spline algorithm. COP data calculated for all time points that the subject was in contact with a force plate COM data synchronized with the COP data to find the maximum horizontal separation distance between the COM and COP in sagittal and coronal planes. First five responses recorded so that “starting position and gait velocity did not factor into the number of answer attempts”	None
Parker et al. (2007) [24]	Eight camera motion capture (Motion Analysis Corporation, Santa Rosa, CA, USA) Thirty-one reflective markers on bony landmarks. Two force plates (Advanced Mechanical Technology, Watertown, MA, USA) positioned in series along gait path.	EVaRT software (Motion Analysis Corporation: Virtual marker positions estimated to represent joint centres and positions of the segmental COM from the external markers. Whole-body COM position calculated as weighted sum of each body segment (13 segments). COM velocities estimated using the generalised cross-validated spline algorithm. One gait cycle: heel strike on the force plate to the next heel strike of the same limb.	None
Parker et al. (2008) [25]	Eight camera motion capture (Motion Analysis Corporation, Santa Rosa, CA, USA) Thirty-one reflective markers on bony landmarks. Marker trajectory data collected at 60 Hz; low-pass filtered using a fourth-order Butterworth filter, cut off frequency 8 Hz. Calibrated prior to each session (volume = 4 m long, 1.5 m wide, 2 m high). Two force plates (Advanced Mechanical Technology, Inc., Watertown, MA, USA) used in series and sampled at 960 Hz.	Whole body COM position data calculated as the weighted sum of each body segment (13 segments). COM velocities and accelerations estimated using the generalized cross-validated spline algorithm. EVaRT software (Version 4.4, Motion Analysis Corpopration): estimating virtual marker positions to represent internal segment endpoints from the external markers, and the relative positions of the segmental centre of mass. One gait cycle: heel strike on the force plate to the next heel strike of the same limb. Data averaged across trials for each task condition (single and dual).	None
Martini et al. (2011) [12]	GAITRite walkway and software 0.89 × 8.3 m with 13,824 sensors embedded recording footfall pressure at 80 Hz. Average performance from five trials for each variable within each condition used for data analysis.	Normalised velocity: velocity/average leg length Step length: heel centre from step to next step. Stride width: distance of foot from midline over two steps. Double support (%): percent of time in gait cycle where both feet on ground. Averaged data from five trials used for analysis.	GAITRite walkway reliability
Fait et al. (2013) [15]	Motion analysis system (Optotrak 3020; NDI, Waterloo, ON, Canada), reflective markers fixed on feet, trunk and head. Data sampled at 100 Hz; low-pass filtered at 6 Hz with a fourth-order zero-lag Butterworth filter. Verbal responses recorded (1000 Hz) with a microphone fixed onto headphones worn by the subject. Pink noise (at 80 dB) played into the headphones to minimise distraction from ambient sounds. Trials videotaped to allow examination of responses to the Stroop word task.	Segment COM estimated by digitising the toe of the shoe, the heads of fifth metatarsal bones, the heels of the shoe, the sternal notch, the lateral surface of the humeral heads, and the ears. Individual scores calculated separately for each dependent variable. For each of the gait conditions, individual averaged scores were means across the five trials. The individual overall pooled scores were means of all gait conditions for each athlete.	None
Howell et al. (2013) [6]	Ten camera motion capture (Motion Analysis Corporation, Santa Rosa, CA, USA) Twenty-nine retroreflective markers on bony landmarks. Data collected at a sampling rate of 60 Hz; marker trajectory data low-pass filtered using a fourth-order Butterworth filter, cut off frequency 8 Hz. Gait events detected from GRFs collected at 960 Hz using three force plates (Advanced Mechanical Technologies, Watertown, MA, USA). Participants verbally responded to the Stroop test using a headset wireless system with microphone (AKG Acoustics, Northridge, CA, USA). For each trial, data were analysed for one gait cycle.	External markers and estimated joint centres used to calculate COM position for each individual body segment. Whole body COM calculated as the weighted sum of all body segments (13 segments). One gait cycle: heel strike to heel strike of the same limb. Average walking speed: mean forward velocity throughout the gait cycle. Step length and step width: distances between right and left heel markers at each heel strike in the AP and ML, respectively. Linear COM velocity: cross-validated spline algorithm from the COM position. Mean of each block of trials for all variables calculated.	None
Cossette et al. (2014) [16]	Nine camera motion analysis system (Vicon, Centennial, CO, USA) recording at 100 Hz. Four triads of reflecting markers placed on subjects’ feet, trunk and head.	Average speed over several strides of the targeted walkway.	None
Howell et al. (2014) [7]	Ten camera motion analysis system (Motion Analysis Corporation, Santa Rosa, CA, USA). Twenty-nine retroreflective markers on bony landmarks. Sampled at 60 Hz; marker trajectory data low-pass filtered using a fourth-order Butterworth filter, cut off frequency 8 Hz. Gait events detected from GRF collected at 960 Hz from three force plates (Advanced Mechanical Technologies Inc., Watertown, MA, USA). Verbal responses recorded using a headset wireless system with a microphone (AKG Acoustics, Northridge, CA, USA).	External markers and estimated joint centres used to calculate COM of each individual body segment. Whole body COM position data then calculated as the weighted sum of all body segments. Linear COM velocity calculated using the cross-validated spline algorithm. Average walking speed calculated as mean forward velocity during gait cycle. Gait cycle: heel strike to heel strike of the same limb. Mean of eight to ten trials for each subject calculated for each variable. Data analysed for one gait cycle.	None
Chen et al. (2015) [28]	Eight camera motion capture (Motion Analysis Corporation, Santa Rosa, CA, USA). 25 retroreflective markers on bony landmarks. Data collected at 60 Hz; marker trajectory data low-pass-filtered using a fourth-order Butterworth filter with the cut off frequency set at 8 Hz. OrthoTrak software (Motion Analysis Corporation) calculated joint angles and gait temporal-distance variables.	Joint angles in sagittal: angular velocities estimated for each joint using the generalized cross-validation spline algorithm. Angular displacements and velocities normalised: phase plots. Data from five trials collected for each testing condition.	None
Howell et al. (2015) [18]	Ten camera motion analysis system (Motion Analysis Corporation, Santa Rosa, CA, USA) at a sampling rate of 60 Hz. Retroreflective markers on bony landmarks. Accelerometer (Opal Sensor, APDM Inc., Portland, OR, USA) attached with an elastic belt at L5. Data sampled at 128 Hz.	Linear acceleration measured along three orthogonal axes, *x* oriented vertically downward, *y* to the right, *z* towards the front. Gait velocity: mean forward velocity of the sacral marker during a gait cycle. Heel strikes used to identify the beginning and the end of the gait cycle. Four trials per subject per testing time point.	None
Cossette et al. (2016) [13]	Motion analysis system (Vicon, Centennial, CO, USA), 100 Hz, used with four triads of reflective markers placed on head, trunk and feet. Low-pass filtered (6 Hz) with a zero lag Butterworth filter.	Specific anatomical references digitized in order to estimate COM positions for the trunk and toe and heel positions for the feet. Gait speed calculated from forward trunk COM movement.	None
Martini et al. (2016) [39]	Spatiotemporal and toe clearance data collected using Vicon (Centennial, CO, USA) system sampling at 240 Hz. Thirty-two reflective markers on bony landmarks.	Gait velocity normalised to height (stature (m)). Step length normalised to height (m).	None
Berkner et al. (2017) [33]	Three inertial sensors (Opal Sensor, APDM Inc., Portland, OR, USA) attached to lumbar spine at lumbosacral junction, and dorsum of each foot with an elastic strap. Data obtained at sampling frequency of 128 Hz.	Gait outcome measures (average gait speed, cadence, stride length, double support time) were calculated using Mobility Lab software (Version 2.0; APDM Inc.).	None
Yasen et al. (2017) [38]	Ten camera motion analysis system (Motion Analysis Corporation, Santa Rosa, CA, USA). Twenty-nine retroreflective markers on bony landmarks. Sampled at 60 Hz; marker trajectory data low-pass filtered using a fourth-order Butterworth filter, cut off frequency 8 Hz. Gait events were detected from ground reaction forces collected at 960 Hz using three force plates (Advanced Mechanical Technologies, Watertown, MA, USA).	External markers and estimated joint centres were used to calculate the centre of mass (COM) of each individual body segment. Whole-body COM position data were then calculated as the weighted sum of all body segments (13 segments). Average walking speed was calculated as the mean forward COM velocity throughout the gait cycle.	None
Howell et al. (2018) [40]	Three inertial sensors (Opal Sensor, APDM Inc., Portland, OR, USA) attached to lumbar spine at lumbosacral junction, and dorsum of each foot with an elastic belt. Data obtained at sampling frequency of 128 Hz.	Gait characteristics calculated using Mobility Lab software (APDM Inc.) (average gait speed, cadence, stride length). Average gait speed: combination of cadence and stride length.	None
Solomito et al. (2018) [20]	Motion data collected at 120 Hz with Vicon motion analysis system (Vicon Motion Systems, Oxford, UK). Sixteen retroreflective markers on bony landmarks. Vicon Nexus used to calculate all temporal and stride parameters. Data filtered using Woltring filter routine found in the Nexus pipeline. Matlab (Mathworks, Natick, MA, USA) used to calculate COM for each stride.	COM calculated by determining the centre point of the upper thoracic plane (C7 and right and left clavicle markers) and the pelvic plane (sacrum and right and left anterior superior iliac spines). COM displacement measured for a total five strides per task, then averaged. COM velocity: time rate of change of displacement determined for each stride and then averaged over the five strides to obtain a single COM velocity value for each task per study participant.	None
Howell et al. (2019) [31]	Opal Sensors (APDM Inc., Portland, OR, USA) attached to lumbosacral junction and dorsum of both feet with elastic strap. Data obtained at sampling frequency of 128 Hz.	Mobility Lab software (ADPM Inc.) calculated gait measures.	None
Howell et al. (2019) [32]	Three inertial measurement sensors (Opal Sensor, APDM Inc., Portland, OR, USA) attached at lumbosacral junction and each dorsum of feet. Sampled at 128 Hz.	Gait variables (average gait speed (m/s), cadence (steps/min), stride length (m)) calculated with Mobility Lab software (ADPM Inc.).	None
Gagne et al. (2021) [41]	Stopwatch	Gait speed estimated as total travelled distance (40 m) divided by total time (seconds) as measured with the stopwatch. DTC for gait speed: % difference between average gait speed during the dual-task and the single-task conditions for the same locomotor task, divided by average single-task gait speed for that same locomotor task. DTC calculated for this variable by subtracting baseline ratio of dual-task ratio, divided by baseline ratio ×100.	None
Martini et al. (2021) [36]	Five inertial sensors (Opal Sensors, APDM Inc., Portland, OR, USA)	Comprehensive gait measures were divided into four domains: pace, rhythm, variability and turning. Domain scores were calculated by averaging the Z-scores for each gait variable. Z-scores were multiplied by −1 to reverse scaling, if needed, for consistent sign in domain score calculations.	43, 44

**Table 3:** Variables and outcome measures in reviewed articles.

Variables	Time Period since Concussion Sustained									
	<72 h		5–7 Days		2 Weeks		1 Month	2 Months	Historic Concussion ^d^	
Gait velocity ^a^	↔ [7,25,28,29]	↓ [9,13,19,23,26,27,41]	↔ [7,13,28,29,33,41]	↓ [9,19,32,34,35]	↔ [7,9,13,28,29,40,41]	↓ [19]	↔ [7,9,13,19,28,29,41]	↔ [7,9,19,41]	↔ [11,12,17,37,38]	↓ [40,42,43]
Stride length ^b^	↔ [7,23,26,27]	↓ [13,25]	↔ [7,13]	↓ [32,35]	↔ [7]	↓ [13,40]	↔ [7,13]	↔ [7]	↔ [12,38]	↓ [40,43]
Stride time	↔ [13,25]	↑ [26,27]	↔ [13]		↔ [13]		↔ [13]		↑ [43]	
Stride width ^c^	↔ [7,13,23,25−27]		↔ [7,13]		↔ [7,13]		↔ [7,13]	↔ [7]	↔ [12,38]	
Cadence			↔ [32]		↓ [35]		↔ [40]		↓ [40]	
Double support %					↔ [40]				↔ [38,40]	↑ [12,43]

Note: ↑ significant increase, ↓ significant decrease, ↔ no significant change, compared to control group. ^a^ includes results for average gait velocity, normalised gait velocity and maximal gait speed. ^b^ includes results for stride length and step length. ^c^ includes results for stride width and step width. ^d^ participants with a history of concussion.

**Table 4:** Reliability articles cited in reviewed articles.

Author (Year) Subjects Referenced by	Protocol Equipment	Variables	Reliability
Paterson et al. (2008) [43] “Younger (Y)” *n* = 13 F 20.08 ± 0.7 y “Older (O)” *n* = 14 F 67.93 ± 7.8 y Reported by Martini et al. (2011) [12]	Two test session days 7 days apart. Single and continuous walking protocols, presented in a random order. Ten walks of 3–5 gait cycles per trial recorded. Two familiarisation trials performed before data collection. Single walking trial: walk along GAITRite at self-selected walking pace. Every second walk was in the opposite position. Continuous walking: curvilinear circuit at preferred speed, walking same direction for each trial. Rest approx. 15 s between trials. Testing in laboratory. Participants wore comfortable walking shoes with a heel less than 2.5 cm. GAITRite 810 × 89 × 0.625 cm. 12 sensor pads, 27,648 sensors placed 1.27 cm apart. 80 Hz.		Inter-Session (Single and continuous trials) Y= younger O = older
		Gait velocity	CV: Y = 4.68, 4.50; O = 4.77, 4.48 ICC: Y = 0.85, 0.81; O = 0.92, 0.93
		Step length (L)	CV: Y = 2.50, 2.06; O = 2.84, 2.47 ICC: Y = 0.94, 0.95; O = 0.94, 0.95
		Step length (R)	CV: Y = 2.56, 2.36; O = 2.61, 2.44 ICC: Y = 0.93, 0.94; O = 0.93, 0.94
		Step time (L)	CV: Y = 2.50, 2.43; O = 3.56, 3.34 ICC: Y = 0.87, 0.86; O = 0.87, 0.87
		Step time (R)	CV: Y = 2.71, 2.21; O = 3.56, 3.78 ICC: Y = 0.87, 0.90; O = 0.86, 0.86
		Step width (L)	CV: Y = NA; O = NA ICC: Y = 0.74, 0.74; O = 0.66, 0.66
		Step width (R)	CV: Y = NA; O = NA ICC: Y = 0.75, 0.71; O = 0.71, 0.70
		Stance time (L)	CV: Y = 3.40, 2.60; O = 3.97, 4.02 ICC: Y = 0.86, 0.90; O = 0.91, 0.90
		Stance time (R)	CV: Y = 3.31, 2.76; O = 3.77, 3.51 ICC: Y = 0.87, 0.89; O = 0.92, 0.92
Montero-Odasso et al. (2009) [42] C *n* = 11, 6 F, 5 M 76.6 ± 7.3 y Diagnosed with mild cognitive impairment Reported by Martini et al. (2011) [12].	ST: walk one length of walkway at a self-selected pace. DT: walk one length while counting backwards from 100 by 1 out loud Testing in a hallway. Three trials per condition per session. Two sessions spaced one week apart. Mean of three trials used for analysis. GAITRite walkway (600 cm long and 64 cm wide)		Inter-Session (Week 1 and 2)
		Gait velocity	CV: ST = 16.96, 13.49; DT = 17.82, 15.63 ICC: ST = 0.87; DT = 0.93
		Step length	CV: ST = 18.26, 16.65; DT = 19.27, 16.21 ICC: ST = 0.97; DT = 0.97
		Stride length	CV: ST = 18.28, 16.51; DT = 19.20, 16.51 ICC: ST = 0.97; DT = 0.97
		Step time	CV: ST = 7.27, 7.02; DT = 11.86; 12.07 ICC: ST = 0.87; DT = 0.96
		Stride time	CV: ST = 6.36, 7.08; DT = 11.02, 11.21 ICC: ST = 0.86; DT = 0.96
		Double support time	CV: ST = 12.90, 12.50; DT = 17.65, 14.71 ICC: ST = 0.80; DT = 0.95
Howell et al. (2017) [9] Subject subset *n* = 28, 17 F, 11 M 19.2 ± 1 y Concussion Classification: History self-reported University athletes Referenced by Howell et al. (2018) [40]; Howell et al. (2019) [32]	Static task: standing static, feet together, hands on hips, eyes open, completing cognitive task for 30 s. ST: walk barefoot at a self-selected pace to a target 8–10 m away, walk around it and return to start. DT: spelling common five letter words in reverse, subtracting by sixes or sevens, reciting months in reverse order. Five trials for each condition. Inertial sensor positioned at lumbosacral junction and dorsum of both feet. Data sampled at 128 Hz. Temporal-distance variables calculated using Mobility Lab (APDM Inc., Portland, OR, USA). Session 1 preseason baseline measures, session 2 conducted 237 ± 53 days following.		ICC ST; DT
		Gait speed	ICC: 0.68; 0.77
		Cadence	ICC: 0.80; 0.85
		Stride length	ICC: 0.71; 0.73
Moore et al. (2017) [44] Stroke patients *n* = 25. 4 F, 19 M 63 ± 11 y Reported by Martini et al. (2021) [36]	Two min continuous walking at a self-selected pace around a 25 m track. Two testing sessions a week apart. 2 weeks continuous usage. Wearable accelerometer (AX3, Axivity, York, UK). GAITRite instrumented walkway (CIR systems, NJ, USA) (7.0 m × 0.6 m) One accelerometer placed on lumbar spine (Opal Sensors, APDM, Inc., Portland, OR, USA). Predefined acceptance ratings for ICCs were set at excellent (≥0.900), good (0.750–0.899), moderate (0.500–0.749) and poor (<0.500).		AX3 vs. GAITRite; AX3 vs. Opal Sensor
		Step velocity	ICC: 0.744; 0.923
		Step length	ICC: −0.411; 0.831
		Step time	ICC: 0.797; 0.890
		Stance time	ICC: 0.758; 0.876
			AX3 test-retest reliability
		Step velocity	ICC: 0.534
		Step length	ICC: 0.419
		Step time	ICC: 0.844
		Stance time	ICC: 0.819
Morris et al. (2019) [45] Young adults *n* = 18, 10 F, 8 M 27 ± 4.4 y Older adults *n* = 18, 10 F, 8M 63.4 ± 9.5 y Parkinson’s disease *n* = 21, 9 F, 12 M 67.5 ± 8.8 y Reported by Martini et al. (2021) [36]	Barefoot walk for 2 min at a self-selected pace walking back and forth over a GAITRite walkway. GAITRite walkway 6 m × 0.6 m. Three inertial sensors (Opal Sensors, APDM, Inc., Portland, OR, USA) placed on both feet and at lumbar spine. Mobility Lab (APDM Inc.) utilised to collect data.		YA; OA; PD; Overall
		Gait velocity	ICC: 0.861; 0.934; 0.920; 0.928
		Stride length	ICC: 0.741; 0.939; 0.880; 0.908
		Cadence	ICC: 0.998; 0.996; 0.996; 0.996
		Stride time	ICC: 0.998; 0.998; 0.992; 0.996
		Double support time	ICC: 0.213; 0.716; 0.285; 0.518

Key: n, number; C, concussed subjects; F, female; M, male; CV, coefficient of variation; ICC, intraclass correlation coefficient; cm, centimetres; m, metres; Hz, hertz; s, seconds; ST, single task; DT, dual task.

## Data Availability

The data in this study is openly available from the journals as listed in the reference section.
